# Intestinal Microbiota and Perspectives of the Use of Meta-Analysis for Comparison of Ulcerative Colitis Studies

**DOI:** 10.3390/jcm10030462

**Published:** 2021-01-26

**Authors:** Ivan Kushkevych, Kristýna Martínková, Monika Vítězová, Simon K.-M. R. Rittmann

**Affiliations:** 1 Department of Experimental Biology, Faculty of Science, Masaryk University, 62500 Brno, Czech Republic; 474593@mail.muni.cz (K.M.); vitezova@sci.muni.cz (M.V.); 2 Archaea Physiology and Biotechnology Group, Department of Functional and Evolutionary Ecology, Universität Wien, 1090 Vienna, Austria

**Keywords:** intestinal microbiome, sulfate-reducing bacteria, hydrogen sulfide, inflammatory bowel diseases, ulcerative colitis, meta-analysis

## Abstract

Meta-analysis is a statistical process summarizing comparable data from a number of scientific papers. The use of meta-analysis in microbiology allows decision-making that has an impact on public health policy. It can happen that the primary researches come to different conclusions, although these are targeted with the same research question. It is, therefore, inevitable to have the means to systematically evaluate information and compare research results. Ulcerative colitis together with Crohn’s disease are among the two main inflammatory bowel diseases. This chronic disease of the gastrointestinal tract, with an as yet unclear etiology, is presented by an uncontrolled inflammatory immune response in genetically predisposed individuals to as yet undefined environmental factors in interaction with the intestinal microbiota itself. In patients with ulcerative colitis (UC), changes in the composition and relative abundance of microorganisms could be observed. Sulfate-reducing bacteria (SRB), which commonly occur in the large intestine as part of the commensal microbiota of animals and humans involved in the pathogenesis of the disease, have been shown to occur. SRB are anaerobic organisms affecting short-chain fatty acid metabolism. This work outlines the perspectives of the use of meta-analysis for UC and changes in the representation of intestinal organisms in these patients.

## 1. Introduction

Ulcerative colitis (UC) is one of the chronic inflammatory diseases of the gastrointestinal tract [1,2]. Its etiology has not yet been fully elucidated [3,4,5]. This disease is presented by an uncontrolled inflammatory immune response in genetically predisposed individuals to as yet undefined environmental factors in interaction with the intestinal microbiota itself [6,7,8]. The importance of the observed changes in the proportion of species of microorganisms in the large intestine in patients with UC is increasingly emphasized by experts [9]. Significant differences in the number of bacteria that penetrated the mucosal barrier into the intestinal mucosa between UC patients and healthy individuals were also confirmed [7,10].

A low number of protective bacteria that produce short-chain fatty acids can lead to increased mucosal permeability. Conversely, enormous concentrations of some bacteria can increase the production of toxic metabolites that increase mucosal permeability and block butyrate metabolism [1]. The large intestine is populated by a specialized group of predominantly gram-negative anaerobes, known as sulfate-reducing bacteria (SRB) [7,11,12,13,14,15,16]. Persistent overproduction of hydrogen sulfide (H_2_S) has been confirmed in patients with active UC, and this fact has been linked to an excess of SRB in the literature [17].

Meta-analysis is a statistical process summarizing comparable data from a large number of scientific papers. It is a quantitative and formal statistical technique that is used for the systematic evaluation of the results of previous research to reach valid conclusions [18,19,20]. Due to the exponential increase in the number of scientific papers dealing with UC issues, it is becoming increasingly difficult to identify relevant information. It is, therefore, necessary to have the means to systematically assess information and compare the results of studies [18].

**Figure 1:**
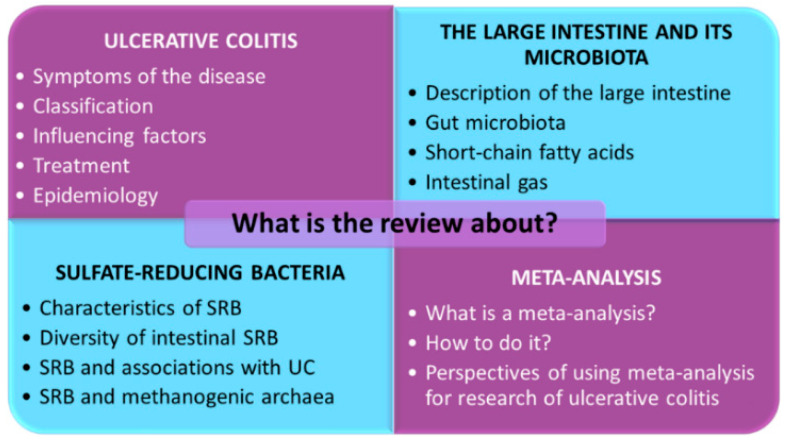
The main points of the review. SRB: Sulfate-Reducing Bacteria; UC: Ulcerative Colitis.

The aims of the research study were to analyze existing information on ulcerative colitis, possible factors influencing the disease, its spread and connection to nutrition, description of the association of intestinal SRB with the development of this idiopathic intestinal inflammation and intestinal microbiota changes as well as to describe a meta-analysis as a method for evaluating these processes and comparing in intestinal microbiota perspectives of using meta-analysis for UC. The main points of the review are shown in Figure 1.

## 2. Ulcerative Colitis

**Figure 2:**
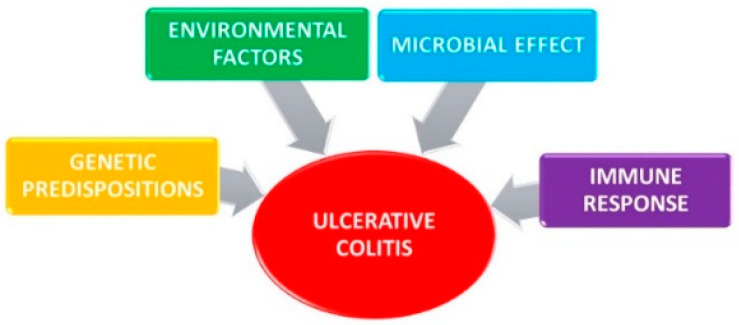
Main factors for the development of ulcerative colitis.

Ulcerative colitis together with Crohn’s disease are the two main inflammatory bowel diseases (IBD) [21,22,23,24,25]. These chronic diseases with an as yet unclear etiology are manifested by an uncontrollable inflammatory immune response to as yet undefined environmental factors in genetically predisposed individuals interacting with their own intestinal microbiota [5,9,26,27]. The main factors for the development of ulcerative colitis are demonstrated in Figure 2.

There are significant differences between the two diseases. Crohn’s disease can affect any part of the gastrointestinal tract, from the oral cavity to the rectum. It most often affects the distal part of the small intestine. UC causes inflammatory changes only in the colon and rectum [28]. UC is manifested by alternating active inflammation with a period of remission [5]. Clinical symptoms of intestinal inflammation include diarrhea, abdominal pain, rectal bleeding, weight loss, and fatigue. Complications of UC include intestinal perforation, where chronic inflammation weakens the intestinal wall to the point that it ruptures. Severe inflammation of the large intestine, caused by the accumulation of intestinal contents, can also result from severe inflammation, including UC. This phenomenon is called a toxic megacolon and is another serious complication [28]. UC may be accompanied by fever or extraintestinal complications such as redness, pain and itching of the eyes, aphthae in the mouth, rash and ulcers on the skin, swelling and joint pain, osteoporosis of the bones, kidney stones, in rare cases also diseases of the liver [28].

Histological findings in patients with UC are characterized by the presence of inflammation, which causes the destruction of mucosal cells. Atrophy of Lieberkühn’s crypts and loss of cryptic architecture are present at all stages of the disease. Neutrophil infiltrations, goblet cell depletion, and crypt abscesses are significant in the active UC. The latter symptom can also be found on the CD [29]. The diagnosis of UC is based on clinical examination, followed by a combination of biochemical, endoscopic, radiological, histological, or nuclear medicine tests. Endoscopy with histology remains the diagnostic standard of common practice [1]. To date, there is no effective treatment to ensure complete recovery of patients with UC, pharmacotherapy, and other procedures only lead to suppression of symptoms and increase the quality of life of the patient [5]. To induce remission, 5-aminosalicylates, corticosteroids, cyclosporine, and the group of monoclonal antibodies infliximab are used (in case of acute severe disease in selected groups of patients). 5-aminosalicylates, azathioprine/mercaptopurine, or methotrexate are administered to maintain remission. If pharmacotherapy fails, surgical removal of the large intestine (colectomy) may be necessary due to the perforation of the intestinal wall, massive bleeding, and also the formation of toxic megacolon [29]. Regular use of probiotics also has a positive effect in the treatment of UC [8].

Pathological findings associated with UC include increased some inflammatory mediators, signs of oxidative stress, impaired microbiome, abnormal glycosaminoglycan (GAG) content in the mucosa, decreased oxidation of short-chain fatty acids (SCFAs), increased intestinal permeability, increased sulfide production, and decreased methylation. Although the primary trigger for UC has not been identified, numerous sub-processes have been elucidated. The interconnection of these findings could lead to clarification of the etiology of the disease described above [30].

### 2.1. Genetic Predisposition

Significant ethnic and geographical differences in incidence and prevalence, the consensus in twins, recurrence in families, and association with genetic syndromes have reinforced the importance of genetics in UC [31].

However, the genetic mechanism involved in the development of the disease has not been fully elucidated. It does not include a single dominant gene or a single recessive gene with full penetration. The most probable mechanism appears to be the combined interaction of several genes, the so-called polygenic or rather multifactorial type of inheritance. Indeed, studies suggest that non-genetic factors (especially environmental or microbial) are able to trigger UC in a genetically susceptible host [32].

UC, like other complex diseases, results from the common effects of polygenic variations [33]. One of the largest meta-analyses in 2012, created by the “International Genetic Consortium for Inflammatory Bowel Diseases” (IIBDGC), has expanded the number of independent risk loci for IBD to 163, much more than is reported for any other complex disease. It turned out that 110 risk loci are important in the pathogenesis of both UC and CD [34].

Risk effects specific for UC only were confirmed in 23 loci and the remaining 30 for CD only. Individual gene variants at many of these loci have not yet been investigated, and even less is known about their functional implications. Interestingly, 43 of these 53 disease-specific loci show the same direction of action in both CD and UC, suggesting that almost all biological mechanisms involved in one disease play a role in the other [31]. One interesting exception is *NOD2*, which is the strongest causal gene for CD production but also shows significant protective effects in the pathogenesis of UC [31,33].

### 2.2. Environmental Factors

Several theories have been proposed to explain unknown environmental exposures that may interact with the immune system and lead to an abnormal inflammatory response to the intestinal microbiome. The most important theory suggests that the increasing frequency of immunological disorders can be attributed to excessive hygiene in childhood and insufficient contact of children with intestinal pathogens. These children are more likely to develop an inappropriate immune response to exposure to new antigens (e.g., gastrointestinal *Helicobacter pylori* infection) later in life [34,35]. Studies have also shown a positive effect of breastfeeding on immunity and its ability to protect against the development of UC at a later age [9].

In terms of lifestyle, there is a higher incidence of UC in people with more sedentary occupations compared to those whose profession requires increased activity [32]. At the same time, psychological stress has been shown to play a role in the etiology and pathogenesis of UC due to the chronic, recurrent and remitting nature of this disease [34]. Both chronic and acute stress can alter immune function. There is evidence of an association between stress, anxiety, and depression with a high risk of developing UC. Depression and anxiety were also associated with a more severe course of the disease and a more frequent need for surgery [9].

The relationship between smoking and UC has also been consistently established. Active smokers are less likely to develop UC than individuals who have never been smokers or are former smokers. However, the exact mechanisms by which smoking affects the development of this disease are not known [34]. A higher incidence of UC was also observed in people who ate more refined carbohydrates in their diet. In addition to increased intake of refined sugars, it was also observed that newly diagnosed patients with UC consumed less fiber, raw fruits, and vegetables compared to healthy controls [9]. The prevalence of UC is largely indirectly related to the prevalence of nematode colonies. Nematodes are thought to play an important immunoregulatory role in the intestinal microbiome. Clinical trials of nematode treatment have shown a potential benefit for UC, which is probably a secondary ability of the parasite to promote immunoregulatory cytokines [34,36].

### 2.3. Microbial Effect

In the large intestine, we find a complex microbial ecosystem. Microorganisms colonizing the large intestine can be found on the surface of the mucosa and also in the feces. The colonial microbiota consists of several hundred microbial species, subspecies, and biotypes [37,38]. There are metabolic relationships between these organisms [38,39].

**Figure 3:**
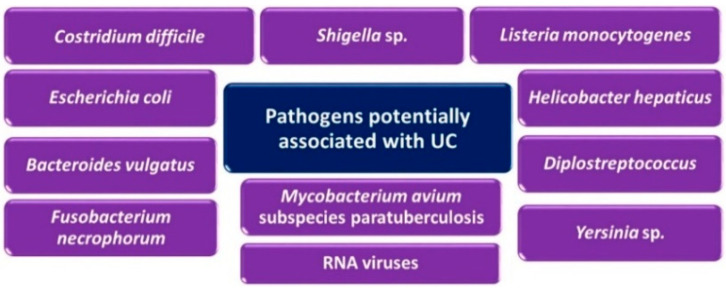
Pathogens potentially associated with the formation or development of ulcerative colitis (UC) [30,40].

Several researchers have confirmed the existence of so-called dysbiosis of the microbial intestinal microbiome in patients with UC. This term refers to changes in the composition and representation of individual species of microorganisms in comparison with healthy individuals. A marked reduction in the incidence of *Firmicutes* (e.g., *Lactobacilli* and *Clostridia*) has been observed. The reduction of the genus *Bifidobacterium* was also observed in the intestinal contents. In contrast, the proportion of *Proteobacteria* (including SRB) and *Actinobacteria* is increasing in patients with UC. Significant differences were found in the number of bacteria that penetrated the mucosal barrier into the intestinal mucosa between patients with UC and healthy individuals [21,22]. Some infectious agents have also been investigated in connection with the pathogenesis of UC. Pathogenic microorganisms potentially associated with the formation or development of UC [30,40]. The pathogens potentially associated with the formation or development of ulcerative colitis are presented in Figure 3.

The unresolved question is whether chronic, recurrent inflammation is caused by persistent infection with a specific pathogen, excessive exposure to normal luminal bacterial products due to increased intestinal permeability, or an abnormally aggressive immune response to luminal components [41].

### 2.4. Immune Response

The main mechanism of inflammatory damage in patients with UC is mediated by an abnormal or exaggerated immune response. HLA (human leucocyte antigen) class II molecules, which mediate autoimmune damage, are found in large numbers within the intestinal epithelial cells of patients with active IBD. HLA class II molecules are responsible for antigen processing and presentation [42]. Activated HLA class II macrophages also secrete elevated levels of proinflammatory cytokines, including interleukins IL-1, IL-6, IL-8, and TNF-*α* within the lamina propria of the intestinal wall. IFN-*γ* is also produced and increases intestinal permeability. The production of IL-2, IL-10, TNF-*β*, and transforming growth factor-beta, which regulates cytokinin levels in patients with IBD, is reduced, and this may explain chronic inflammation in patients [9].

In the absence or after the functional loss of these anti-inflammatory feedback signals, physiological defense mechanisms may turn into pathological responses. The data suggest that disorders in homeostasis between signals obtained from bacteria and hosts at the epithelial cell level lead to disruption of intestinal barrier function and thus the development of mucosal immune disorders in genetically sensitive hosts [43].

### 2.5. Epidemiology

**Figure 4:**
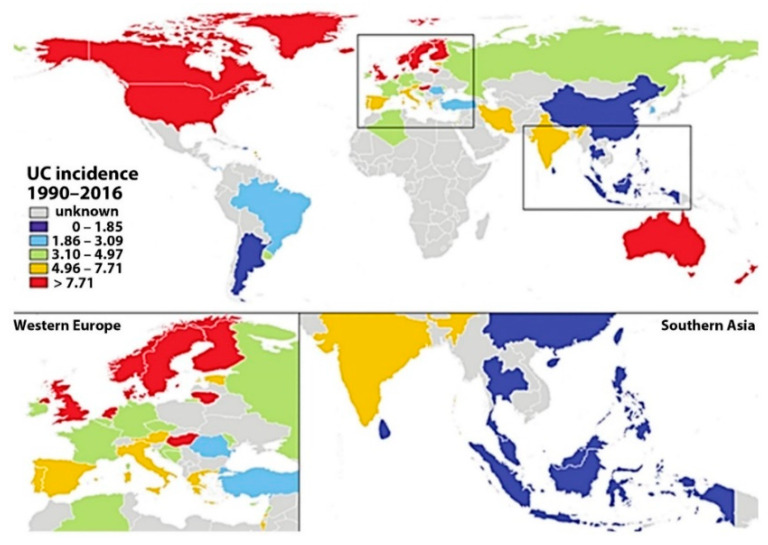
Ulcerative colitis (UC) incidence map between 1990–2016 [44].

UC has an annual incidence of 10–20 per 100,000, but these data are generally considered underestimated. It mainly affects the younger population with a maximum incidence between 20 and 40 years but can affect any age group and up to 15% of individuals are over 60 years of age at the time of diagnosis. It is currently estimated that IBD affects up to 1.4 million people in the United States and 2.2 million in Europe. Gender differences have been observed in patients with a slight predominance of men [1]. Epidemiological studies have shown a north-south gradient with a higher incidence of UC in the Scandinavian countries and a lower one in the Mediterranean. In recent years, the west-eastern gradient with the highest incidence of UC on the Atlantic coast of Europe and the lowest in Asian areas has also been confirmed [33]. Ulcerative colitis (UC) incidence map between 1990–2016 is presented in Figure 4.

The incidence of this disease in the Czech Republic is between 12–15 patients per 100,000 population, i.e., the mean value between Western and Eastern Europe. However, the available data show a high increase in the total number of patients treated. In the case of the Czech Republic, the number of outpatients increased from about 20,000 in 2007 to 24,000 in 2013. There was also an increase in the number of newly diagnosed cases from the number of newly diagnosed cases in the monitored period 1700 to 2500. The number of deaths with the main diagnosis of UC in the Czech Republic varies from 26 to 50 cases during each year [1].

## 3. The Large Intestine and Its Microbiota

The large intestine (intestinum crassum) is the last part of the digestive tract ensuring the resorption of water, amino acids, bile acids, salts, vitamins and removes unabsorbed residues such as feces. This organ is 1.3–1.4 m long and 5–8 cm wide. The main sections of the large intestine are the caecum from which the appendix vermiformis, the colon, the rectum, and the anal canal analis extend. The intestinal wall consists of mucosa, mucosal ligament, muscle, and serosis.

The mucosa of the large intestine is smooth, slightly articulated. Kerckring’s algae and intestinal villi, which are found in the small intestine, are absent in the large intestine. Lieberkühn’s crypts have a tubular appearance and are significantly longer than in the small intestine. The epithelium of the colonic mucosa is single-layered cylindrical and consists of enterocytes and goblet cells, which are particularly abundant in the middle sections of Lieberkühn’s crypts. Paneth cells are usually missing. Of the endocrine cells, the epithelium contains mainly EC cells (so-called enterochromaffin cells), which, among other things, synthesize serotonin. The intestinal epithelium is a physical barrier that coexists with intestinal microorganisms, ensuring the transport of substances and their regulation so that homeostasis is maintained [45].

### 3.1. Intestinal Microbiota

There are approximately 10^13^–10^14^ bacteria in the human gut, with about 10^13^ cells in the human body [46,47]. Thus, there are as many or up to ten times more bacteria in the human body than human cells alone [38,39]. Bacteria in the large intestine are thought to make up almost 90% of the total human colonic microbiota [48].

Profiling of the 16S rRNA gene sequence showed that bacteria of the strains *Bacteroidetes*, *Firmicutes*, *Actinobacteria*, *Proteobacteria*, *Verrucomicrobia*, and also representatives of the domain *Archaea* predominate. Bacterial strains of *Cyanobacteria*, *Fusobacteria* and *Spirochaete* are also present in the intestines, but in smaller numbers. The microbiota has an important metabolic, immunological, and protective function [49].

The composition of the microbiome is therefore crucial because billions of bacterial individuals in the intestinal lumen can become a threat to the host organism with any change in conditions. Although metabolites produced by the microbiota affect various organs and systems in the body by signaling, symbiosis with the intestinal mucosa is necessary for their survival [50]. The main representatives of intestinal bacteria are presented in Table 1.

The human body has well-developed mechanisms by which it effectively prevents the translocation of symbiotic or pathogenic microorganisms across the mucosal barrier, including mucus protection of the intestinal epithelium. Intestinal mucus has a highly organized glycoprotein network structure but also contains a stable proteome [51]. Intestinal epithelial cells separate the intestinal lumen and deeper tissue structures that normally contain immune cells [52,53]. By contacting or penetrating the epithelial barrier, microorganisms affect immune cells and can cause the induction of an immune response [54].

Anaerobic colon bacteria break down carbohydrates and proteins through fermentation into gases and SCFAs [55], which are a major source of energy for the colon epithelium [52]. Saccharolytic bacterial fermentation produces generally beneficial metabolites. However, while under carbohydrate-limiting conditions, bacteria turn to alternative energy sources, leading to the production of other metabolites that might be harmful to human health [53].

Indigestible polysaccharides present in, for example, fruits and vegetables pass without effective cleavage to the large intestine, where they are fermented to form SCFAs as products of bacterial metabolism [54]. Low intake of indigestible polysaccharides results in reduced levels of SCFAs and their producers. An interesting finding was the fact that increased intake of fruits and vegetables is associated with a reduced risk of developing both UC and CD, and SCFAs could, therefore, be good candidates for deeper research into the regulation of the immune response under the influence of various inducing factors [56]. Decreased levels of SCFAs have been confirmed in children with IBD [57].

The intestinal microbiota also has the significant proteolytic capacity, converting ingested food protein and endogenous protein from host enzymes and mucin to shorter peptides, amino acids and their derivatives, short and branched-chain fatty acids, and gases, including CH_4_, NH_3_, H_2_, CO_2_, and H_2_S [7,53]. *Bacteroides* and *Propionibacterium* are the dominant proteolytic species in fecal samples, with proteolysis also being common to *Clostridia*, *Streptococci*, *Staphylococci*, and *Bacillus* species [10,53].

Colon bacteria are also able to synthesize some vitamins, especially B vitamins, including biotin, cobalamin, folate, nicotinic acid, pantothenic acid, pyridoxine, riboflavin, and thiamine, and vitamin K [53,58]. One of the first documented benefits of intestinal commensals for human metabolism was undoubtedly their confirmed ability to produce vitamin B_12_. These vitamins are important for both bacterial metabolism and the host [59].

### 3.2. Short-Chain Fatty Acids

Indigestible polysaccharides, including cellulose and other substances, are fermented in the gut by anaerobic bacteria to obtain energy for microbial growth and production of short-chain fatty acids (SCFAs) [60]. The three most abundant SCFAs detected in feces are acetate, propionate, and butyrate [61].

Butyrate is important representative of SCFAs and plays a key role in maintaining intestinal epithelial homeostasis as a preferred source of energy for colonocytes and their growth stimulator [62]. Previously published studies have found that 71% of the energy obtained by colonocytes is due to butyrate, which is preferentially produced by commensals, especially representatives of *Clostridia* sp. from the *Firmicutes* phylum strain [63]. It is also used as an inhibitor of carcinogenesis, inflammation, and oxidative stress that stimulates mucus production and absorption of electrolytes and fluids and improves the barrier function of the intestinal epithelium [62].

Butyrate also has anti-inflammatory effects by stimulating antigen-presenting cells to produce the cytokines TGF-*β*, IL-10, IL-18, and by inducing the differentiation of naive T cells into regulatory T cells. A mild anti-inflammatory effect was also demonstrated for acetate and propionate. These two molecules have the ability to suppress the production of proinflammatory cytokines by stimulating TLR 4 [64].

Butyrate metabolism is one of the current theories for the etiology of UC [65]. It is thought that a lack of energy for colonocytes could lead to the onset of this disease [41,52]. Butyrate has also been used experimentally in the treatment of colitis with numerous benefits for patients [54,66].

Propionate, like butyrate, can induce T-cell differentiation [64]. Propionate is a source of energy for epithelial cells, but it is also transferred to the liver, where it plays a vital role in gluconeogenesis (IGN). A study in mouse models showed that after infusion of the propionate solution, 62% of this substance was used as a substrate for IGN throughout the body of the experimental animal. Glucose synthesis from propionate represents 69% of the total IGN in the body [67]. It is also increasingly considered an important molecule in satiety signaling due to interaction with the intestinal GPR 41 and GPR 43 receptors [53,68], also known as FFAR2 and FFAR3 fatty acid receptors, which may, in turn, activate intestinal IGN [53,69].

Acetate is the most abundant SCFA and is an important cofactor and metabolite for the growth of many microorganisms [61,70]. In the human body, acetate is transported to peripheral tissues and is taken up by cholesterol metabolism and lipogenesis. Recent studies expect them to play a significant role in the central regulation of appetite [71]. SCFAs are demonstrably connecting the links between dietary routines, intestinal microbiota, and host energy metabolism [67]. SCFAs are advantageous energy coverage of up to 10% of the daily caloric potential of the human body [63].

Bacterial fermentation provides numerous intermediates, including fumarate, succinate, and lactate, but are commonly detected at low levels in the feces of healthy individuals due to their extensive use by other bacteria. For example, lactate is usually converted to propionate or butyrate by other bacteria and is therefore present at negligible levels in adult feces. However, in patients with UC, lactate can be detected in significantly higher amounts and could be a potential indicator of the disease [53,72]. It has been shown that the control and manipulation of the intestinal microbiome can be an approach to the therapy and prevention of IBD [7].

### 3.3. Intestinal Gases

The gas is a product of microbial fermentation in anaerobic ecosystems, including the digestive tract. However, some anaerobically growing species produce no gas [53,73]. This is the case with common probiotics such as *Lactobacilli* and *Bifidobacteria*. It is therefore theoretically possible that probiotics or prebiotics can reduce the occurrence of gas in the intestines and also contribute to the suppression of some health problems [10].

Most microbially generated gas includes CH_4_, NH_3_, H_2_, CO_2_, and H_2_S, among others. The key harmful and potentially toxic components are sulfides, which act as precursors to other sulfur-based components [7]. However, sulfide is also an important signaling molecule for bacteria, plants, and mammals. In the human body, sulfide acts as a messenger for a variety of systems, including the central and peripheral nervous systems, the immune system, and the gastrointestinal tract. Sulfide gas enters and also leaves cells due to diffusion across the lipid membrane [74].

The H_2_ composition of the flatus is up to 40% and appears to be exclusively of microbial origin [10]. H_2_ is produced by various intestinal microorganisms. The main producers are *Bacteroides* and *Clostridium*. There are three main microbial pathways by which H_2_ can be removed. These include sulfate dissimilation reduction, methanogenesis, and acetogenesis [75].

The dissimilative reduction of sulfates is provided by SRB. These microorganisms use sulfate as an electron acceptor to dissimilation organic compounds and H_2_ [76]. The most common genus SRB in the intestine is the genus *Desulfovibrio*. Sulfate can be ingested in the diet or released after microbial metabolism of sulfate mucins. These are glycoproteins that line the gastrointestinal tract and act as a protective barrier between the mucosal surface and the luminal contents [77].

The use of H_2_ to reduce sulfate to sulfide has effects on overall gas production in the colon by reducing the amount of free H_2_, thereby helping to prevent excessive gas accumulation. However, the highly toxic nature of the H_2_S produced can have pathological consequences for the host [53]. The SRB group is described in more detail in Chapter 4.

Dissimilatory sulfate reduction: 4H_2_ + SO_4_^2−^ + H^+^ → HS^−^ + 4H_2_O

Methanogenesis is another mechanism of H_2_ removal in the large intestine that also reduces the overall gas accumulation. Thus, methanogenic archaea and SRB compete for H_2_ in the gut, and the process that dominates depends on the amount of sulfate available. When sufficient sulfate is available, SRB are more easily recovered by H_2_ due to their higher affinity for the substrate [10,53].

Methanogenesis: 4H_2_ + CO_2_ → CH_4_ + 2H_2_O

For host health, acetogenesis is probably the most preferred way to recycle H_2_. The reason is the conversion of carbon dioxide (CO_2_) and H_2_ into acetate without releasing gas. However, this reaction is less energetically favorable than reduction by dissolving sulfate or methanogenesis [75].

Acetogenesis: 4H_2_ + CO_2_ → CH_3_COOH + 2H_2_O

CO_2_ is another quantitatively significant gas. CO_2_ can represent between 5% and 50% of the total volume of the flatus and is recycled by methanogenesis and to a lesser extent by acetogenesis. Unlike H_2_ and methane, CO_2_ can be generated by a number of processes, not just bacterial metabolism [78].

The absence of SRB, methanogens, and acetogens would cause the individual to produce 5–10 times more gas than usual. H_2_ recycling by disulfilization reduction of sulfate generates H_2_S, which is a cellular signaling molecule, but also a highly toxic substance for colon cells, and its production and amount is a cofactor of inflammatory bowel disease. The presence of methane in the colon has been linked to colorectal cancer, although this association may be due to disease rather than causation, as patients with these difficulties also suffer from slower intestinal passage through the colon, which benefits the growth of intestinal methanogens due to their slowly growing nature [53].

## 4. Sulfate-Reducing Bacteria

Sulfate-reducing bacteria (SRB) are common in anaerobic areas of soil, wetlands, fresh and seawater, but also the colon of humans and animals or the oral cavity. These microorganisms dissimilate sulfate to H_2_S by a process called sulfate dissimilation reduction and participate in the process of the biogeochemical cycle of sulfur in nature [79,80]. These are strictly anaerobic microorganisms present in anoxic environments rich in sulfates [81,82,83,84]. SRB can use lactate, pyruvate, formate, acetate, propionate, butyrate, ethanol, fructose, acetone, dicarboxylic acids, and amino acids as carbon and energy sources [80]. This method of nutrition is called chemo-organoheterotrophy. In addition to these compounds, SRB can sometimes utilize CO_2_, which may be the only carbon source for autotrophic growth [79].

H_2_S produced by the reduction of sulfates is highly toxic to all living organisms, so it must be removed from the body, which is ensured by various mechanisms, including oxidation by microorganisms [85,86]. It should be mentioned that H_2_S is toxic not only to the host but also to its bacterial producers themselves. High concentrations (above 6 mmol) of H_2_S lead to the cessation of SRB growth and the development of inflammatory damage to the intestinal epithelium [3,86].

A diet rich in sulfate ions, such as foods preserved with sulfur oxides, causes an increase in the concentration of H_2_S produced by SRB. It has been reported that the western diet contains more than 16.6 mmol of sulfates per day and the feces of approximately half of healthy individuals contain SRB, with 92% of the genus *Desulfovibrio* predominating [10]. Intestinal SRB include the genera *Desulfotomaculum*, *Desulfobulbus*, *Desulfomicrobium*, *Desulfomonas*, and *Desulfovibrio*. The dominant species of SRB in the human intestinal tract is *Desulfovibrio* sp. [22,25,87,88]. SRB are not thought to be pathogenic in humans and animals. However, together with other infections, they can cause various diseases. The main way SRB penetrates blood vessels is probably damage to the intestinal mucosa and then bacteria causing infection [10]. Previously published work provides information on the relationship between the presence of SRB in the human gut and the prevalence of cholecystitis, encephalitis, and UC [22,85]. SRB form a biofilm in the gut together with other bacterial species such as *Bacteroides*, *Pseudomonas*, *Clostridium*, and *Escherichia* [13].

SRB grow on the surface of the mucosa or in the intestinal lumen. Living bacteria on the surface of the colonic mucosa, they interact with cells of the immune and neuroendocrine systems more closely than microorganisms in the intestinal lumen [10]. The species composition and number of SRB on the surface of the intestinal mucosa differ from the microorganisms in the feces. The presence of sulfate ions promotes the growth of intestinal SRB that use H_2_ and compete for this substrate with methanogenic archaea [6]. The prevalence of SRB varies depending on the individual. Species of the genus *Desulfovibrio* represent 67–91% of the total number of SRB. Significantly fewer bacteria occur in the genera *Desulfobacter* (9–16%), *Desulfobulbus* (5–8%), and *Desulfotomaculum* (2%) [89]. Examples of representatives of the group of sulfate-reducing organisms are presented in Table 2.

### 4.1. Diversity of Intestinal Sulfate-Reducing Bacteria

#### 4.1.1. Order *Desulfovibrionales* (Domain *Bacteria*, Phylum *Proteobacteria*, Class *Deltaproteobacteria*)

Bacteria of the order *Desulfovibrionales* are straight or curved, motile rods. In relation to molecular oxygen (O_2_), they are strictly anaerobic. These bacteria are mesophilic or slightly thermophilic. They grow under neutral to slightly alkaline conditions. Sulfide, sulfide, and thiosulphate are reduced to H_2_S [91].

#### 4.1.2. Family *Desulfovibrionaceae*, Genus *Desulfovibrio*

Bacteria from the *Desulfovibrionaceae* family oxidize organic matter to acetate. In addition to the gastrointestinal tract and feces, they can also be found in the genitals, oral cavity, and in the aquatic environment. Representatives of the genus *Desulfovibrio* are G^−^, curved, or spiral cells, mostly motile. The electron donor is usually H_2_, lactate, ethanol. The optimal temperature for growth is between 25 and 40 °C [91].

#### 4.1.3. Family *Desulfomicrobiaceae*, Genus *Desulfomicrobium*

The genus *Desulfomicrobium* includes ovoid, rod-shaped G^−^ cells, motile or immobile. They oxidize H_2_, lactate, and malate. Organic substrates are incompletely oxidized to acetate. By type, representatives are difficult to distinguish. In addition to settlements in the intestine, they also occur in O_2_-free freshwater, in marine sediments, or water from oil fields [79,91].

#### 4.1.4. Order *Desulfobacterales* (Domain *Bacteria*, Phylum *Proteobacteria*, Class *Deltaproteobacteria*)

The morphology of bacteria of the order *Desulfobacterales* is diverse. They can form cocci or oval to regular rods, which can be curved. Sometimes we find them in clusters or threads. They are often mobile. Strictly anaerobic. This order also reduces sulfate, sulfide, and thiosine to H_2_S [91].

#### 4.1.5. Family *Desulfobacteraceae*, Genus *Desulfobacter*

These bacteria are capable of complete oxidation of organic substrates. Representatives of the genus *Desulfobacter* are G^−^. The cells are oval or rod-shaped, slightly curved, movable, and immobile. The donor of electrons is most often acetate, but it can also be ethanol, H_2_, and lactate [79,91].

#### 4.1.6. Family *Desulfobulbaceae*, Genus *Desulfobulbus*

The *Desulfobulbaceae* family includes cocci and rods, which are often motile. Simple organic substances serve as an electron donor and at the same time as a carbon source. They incompletely oxidize substrates and the final product is usually acetate. Mesophilic and psychrophilic organisms belong to the family *Desulfobulbaceae*. Representatives of the genus *Desulfobulbus* are G^−^ type, mostly in the shape of an ovoid rod. The electron donor is propionate, but they also oxidize H_2_, lactate, ethanol [91].

#### 4.1.7. Order *Clostridiales* (Domain *Bacteria*, Phylum *Firmicutes*, Class *Clostridia*)

Includes genus *Clostridium* and related genera. Anaerobic G^+^ bacteria with low G+C content in DNA [91].

#### 4.1.8. Family *Peptococcaceae*, Genus *Desulfotomaculum*

The *Peptococcaceae* family includes cocci and sticks. We can also find saprophytic or clinically important species here. Representatives of the genus *Desulfotomaculum* are straight or curved rods, movable, and occurring individually. These bacteria form round to oval endospores, terminally or subterminally deposited, swollen cells. They are the only intestinal SRB that form spores. They grow at a temperature of 20–70 °C [91].

### 4.2. Sulfate-Reducing Bacteria and Ulcerative Colitis

Decreased concentrations of protective bacteria that produce short-chain fatty acids (SCFAs), such as butyrate, may increase mucosal permeability. Conversely, elevated concentrations of some bacteria may increase the production of toxic metabolites, such as H_2_S, which increases mucosal permeability and block butyrate metabolism. Increased mucosal permeability can lead to the activation of pathogenic T cell-mediated and innate immune responses through exposure to bacterial TLR ligands and antigen [1].

In the large intestine a specialized group of predominantly gram-negative anaerobes is present, known as SRB, which are capable of a process called the reduction of dissolving sulfates, reducing sulfates to sulfides. Sulfide is toxic to the intestinal epithelium. Thus, sulfate in the diet can selectively stimulate the growth of one group of bacteria with potentially harmful effects on the epithelium [7].

**Figure 5:**
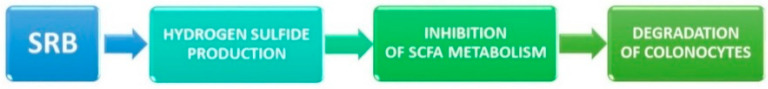
Mechanism of H_2_S toxicity. SRB: Sulfate-Reducing Bacteria.

Patients with active UC overproduce H_2_S, toxic to the intestinal mucosa by competition with SCFAs, which appears to be associated with an excess of SRB (e.g., *D. desulfuricans*) in fecal samples [17]. The estimated number of SRB and the level of H_2_S accumulation in human feces may predict the course of inflammation in the gut [30]. New opportunities to study IBD and assess the effectiveness of its treatment are extremely pressing topics in modern biology and medicine [80]. The mechanism of H_2_S toxicity is presented in Figure 5.

### 4.3. Sulfate-Reducing Bacteria in Relation to Methanogenic Archaea

H_2_ is the only electron donor for intestinal methanogenic archaea such as *M. smithii*. Therefore, there is competition for H_2_ between SRB and methanogenic organisms. When sulfates are present in sufficient amounts, SRB inhibit the use of H_2_ by methanogens in the dissimilation of the sulfate reduction process. Thus, the amount of sulfate in the diet may affect the competition of the substrate between SRB and methanogensin the large intestine. In the absence of sulfate in the diet, SRB were not found. Thus, the intensity of methanogenesis can be regulated by the introduction of sulfate, even when the SRB are low in the gut [6,10]. The most common substrates for SRB in the human colon are lactate, pyruvate, acetate, and ethanol, which can be electron donors in the process of reducing dissolving sulfate [82].

## 5. Meta-Analysis

Meta-analysis is a statistical process summarizing comparable data from a large number of scientific papers [19,20,21]. This is a quantitative and formal statistical technique that is available for the systematic evaluation of research results to reach a valid conclusion [92]. The results of a meta-analysis may be more accurate estimates of treatment or disease risk factor or outcome results than your studies that contribute to the joint analysis and is therefore considered the top of the hierarchy of scientific evidence assessment [92]. The meta-analysis makes it possible to evaluate the results of studies and the possible risks of their misinterpretation [93].

Due to the exponential growth of scientific studies, it is becoming increasingly difficult to identify relevant information. It happens that the primary studies bring different conclusions, although they address the same research question [94]. Meta-analyzes are used in microbiology to address key research issues, such as the prevalence of pathogens and the incidence of infectious diseases, and can facilitate public health decision-making. Research to estimate the use of this tool in microbiology indicates a significant increase in the use of this research methodology since 2007. This method is most widely used in Europe and North America [94].

### 5.1. Performing a Meta-Analysis

#### 5.1.1. Literature Research and Data Abstraction

Proper meta-analysis is characterized by a thorough and disciplined search of the literature and, last but not least, discussions with experts in the field. A clear definition of the hypotheses to be investigated is important [93]. For studies in the field of microbiology, meeting these conditions means ensuring a sufficient number of results of primary studies that address, for example, the relation of key risk factors (often pathogens) with the disease [94].

The “Preferred Reporting Items for Systematic Reviews and Meta-Analyzes” (PRISMA) statement recommends a thorough search of key sources from at least one major database [93]. There should still be a typical strategy for searching all available literature, which includes the scientific databases “PubMed” (http://www.ncbi.nlm.nih.gov/pubmed), “ScienceDirect” (www.sciencedirect.com), “Institute of Scientific Information Web of Knowledge” (http://www.isiwebofknowledge.com) and “Google Scholar” (http://scholar.google.com) [95]. Complex researches usually lead to a large number of identified records, to a much smaller number of studies included in the systematic survey, and even fewer of these studies included in the meta-analysis. From the keywords found, studies based on an abstract are excluded, followed by a full-text examination and the use of eligibility criteria. The authors of the publication often state the initial number of records identified and the number of studies included. Ideally, the reasons for excluding studies should be given as text in combination with the use of a flowchart [93].

#### 5.1.2. Forest Plot

**Figure 6:**
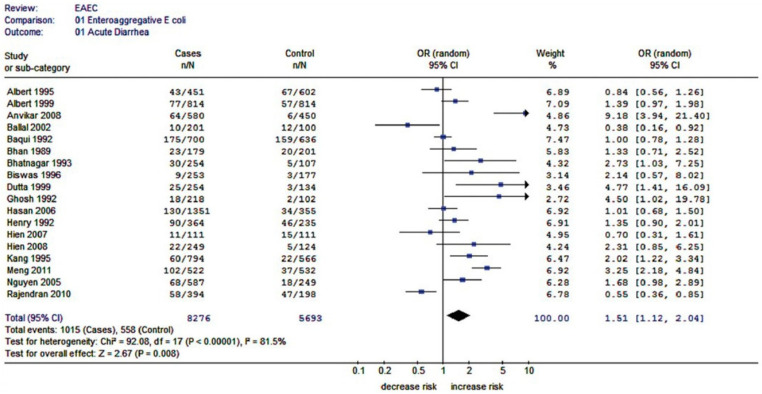
Illustrative image of forest plot generated by RevMan [94].

The meta-analysis presents knowledge about the size of the tested file, which makes it possible to identify connections between individual information and draw new conclusions. In the meta-analysis example, the results of each study are graphically presented in a forest plot, a method that uses an odds ratio (OR) and a confidence interval (CI) metric of 95% [94]. Forest plot (you can also find the less used Czech name “Forest graph”) can be generated by some of the software for meta-analysis (e.g., RevMan or SigmaPlot). When generated in RevMan, it typically consists of six columns, with the leftmost qualitative data (studies named after the first author’s last name) and the remaining four providing quantitative data. Between columns three and four, there is a forest fence with a fixed vertical line (marked 1 on the x-axis), which corresponds to the zero effect. The area to the left of the vertical line indicates reduced risk and the right side reflects increased risk of IBD. The two columns on the far left show the raw data (cases/controls) and a 95% CI is calculated (the far-right column). The diamond (♦) at the bottom of the forest fence is a summary of the effect [94]. Illustrative image of forest plot generated by RevMan is presented in Figure 6.

There are many ways to perform a meta-analysis. Commonly used statistical models for combining data in the meta-analysis are the fixed-effect model and the random-effect model. The fixed-effect model assumes that there is a common effect in all included studies and that the observed differences in results across the studies reflect random changes. The random-effects model assumes that there is no specific effect on all studies. The fixed-effect model only considers heterogeneity within the study, while the random effect model takes into account heterogeneity within and between studies [93].

## 6. Perspectives of Using Meta-Analysis for Ulcerative Colitis

Meta-analysis is usually used for topics that do not have definitive studies or do not completely agree with the conclusions of these studies. Studies with little or no statistical power to confirm a fact often appear. By combining the results of several studies, sufficient statistical power can be achieved, and thus new facts can be discovered (e.g., the actual effect of treatment) [96]. UC is a complex disease, while connecting a large number of scientific areas. Studies using meta-analysis were sought for this chapter and address the issue of UC. Perspectives of using meta-analysis for research of ulcerative colitis are presented in Table 3.

### 6.1. Sulfate-Reducing Bacteria and Probiotic Treatment of Ulcerative Colitis

CD can affect any part of the gastrointestinal tract. UC is limited to the large intestine and rectum [28]. This may be due to the acidic pH of the stomach (unfavorable environment for SRB), while the large intestine has a pH lower than 5.5, and in the distal part of the large intestine, the pH is neutral, which is considered the optimal condition for SRB growth [10,80]. SRB are not directly pathogenic to humans and animals [23,97], yet today they are considered one of the potential factors that support the development of IBD [27]. A diet with a higher sulfate content can significantly affect the number of SRB in the intestines and the composition of the intestinal microbiota. The sulfate content above 8 μmol g^−1^ can be found in dried fruits and vegetables, in nuts (especially almonds and hazelnuts), cruciferous vegetables (broccoli, cabbage, brussels sprouts), in white and dark bread, soy flour and sausages [77].

**Figure 7:**
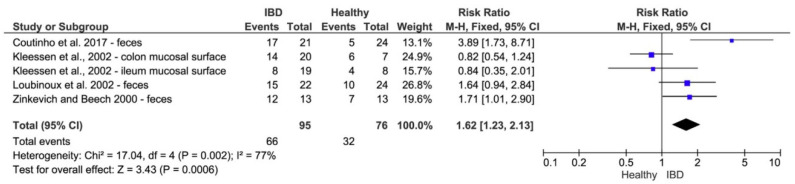
Forest plot showing the relation between the presence of sulfate-reducing bacteria (SRB) and the prevalence of inflammatory bowel diseases (IBD) [21].

**Figure 8:**
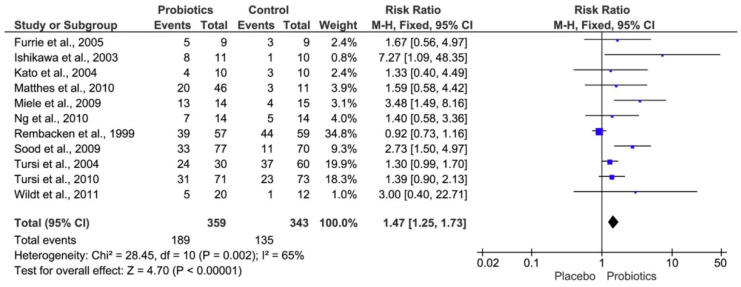
Forest plot showing the effect of probiotics on inflammatory bowel diseases (IBD) [21].

A comprehensive overview of the properties of intestinal SRB and their relationship to IBD was reported in the study published in 2020: “Hydrogen sulfide toxicity in the gut environment: meta-analysis of sulfate-reducing and lactic acid bacteria in inflammatory processes”. This study also highlights the positive effect of probiotics. A meta-analysis was used for statistical comparison of the respective studies. The meta-analysis included 16 studies, which were selected from the Web of Science database. RevMan software was used to create the meta-analysis. The meta-analysis shown in Figure 7 includes studies on the relationship between SRB and IBD and the meta-analysis in Figure 8 the relationship between probiotic lactic acid bacteria (LAB) and IBD [21].

All studies except one found a significant (*p* < 0.05) difference between the incidence of SRB in healthy people and patients with advanced IBD. However, diamond, which summarizes all studies, suggests the occurrence of SRB mainly in people with IBD [21]. The treatment of patients with UC with probiotics is shown in Figure 8.

The studies were conducted in such a way that the group was always divided into a control group and a probiotic group. Comparative studies show that most of them touch the zero-effect line and therefore have no significant benefit, except for the study by Ishikawa et al. (2003) [98], Miele et al. (2009) [99], and Sood et al. (2009) [100], where a significant (*p* < 0.05) difference was noted. All studies except the study of Rembacken et al. (1999) [101] show that the use of probiotics affects the course of IBD and the symptoms of the disease were reduced during use. Diamond, which summarizes all studies, suggests that the administration of probiotics to patients with IBD has a positive effect on the symptoms of the disease [21]. A lower incidence of IBD was confirmed within the group of subjects receiving probiotics and at the same time a higher prevalence of IBD in patients with a higher incidence of SRB.

### 6.2. Treatment of Ulcerative Colitis with Infliximab

Tumor necrosis factor-alpha (TNF-*α*) is currently one of the most important drug target molecules used in several autoimmune diseases. Infliximab became the first TNF-α-inhibiting drug to be introduced into clinical practice. At the same time, it was the first registered biotechnologically prepared drug with the character of a monoclonal antibody. It is used in patients with IBD, but also a number of other autoimmune diseases (e.g., rheumatoid arthritis or Bechterew’s disease). It can reduce the severity of the symptoms of the disease and cause remission [102]. It has been confirmed that after infliximab administration, the intestinal mucosa is restored in patients [103]. Infliximab appears to be a safe and effective alternative treatment and its application has become the most important event in the treatment of UC in the last 10 years [104]. As many as 25% of UC patients experience at least one acute severe episode during their lifetime that requires hospitalization [105]. If pharmacotherapy fails, colectomy may be necessary in approximately 40% of acute severe cases. Emergency colectomy still carries significant risk, almost 10% mortality within 3 months of surgery (data from 2006) [29,106].

**Figure 9:**
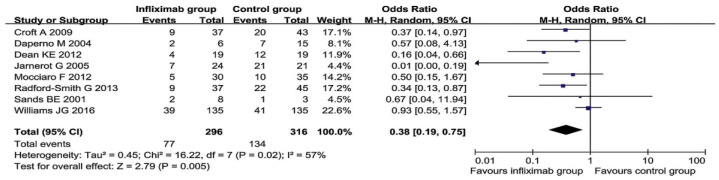
Forest plot of colectomy rates in the infliximab group of patients for 3 months and in the control group [104].

**Figure 10:**
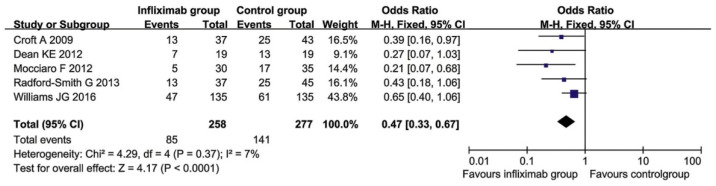
Forest plot of colectomy rates in the infliximab group for 12 months and in the control group [104].

A systematic review from 2019 “In infliximab clinically treating ulcerative colitis: a systematic review and meta-analysis” is an evaluation of current UC treatment. This meta-analysis was performed in accordance with PRISMA criteria. All statistical analyzes were performed using RevMan 5.3 software. For a meta-analysis, studies comparing the efficacy of infliximab versus placebo, steroids, or immunosuppressants with UC were included. Only studies with clear information on the number of patients who were included and grouped for each therapy [104]. The rate of colectomy was observed in eight studies reporting a rate of colectomy after 3 months and five studies on the frequency of colectomy after 12 months. Among the studies in the meta-analysis of the colectomy rate after 3 months of treatment, heterogeneity was found (*p* = 0.02), but no heterogeneity was found in the colectomy rate after 12 months of treatment. Therefore, the method of random effects was used for the colectomy rate after 3 months, and the model of fixated effects for the colectomy rate after 12 months. A meta-analysis showed that the colectomy rate in the infliximab group was significantly lower than in the control group after 3 and 12 months of treatment, as shown in Figure 9 and Figure 10 [104].

This meta-analysis showed that infliximab reduced the rate of colon colectomy in patients with moderate to severe UC at 3 and 12 months. Additional meta-analyses were performed in the study to confirm that infliximab administration promotes the onset of remission compared to traditional drugs or placebo [104].

### 6.3. Clostridium difficile and Ulcerative Colitis

*Clostridium difficile* (domain *Bacteria*, strain *Firmicutes*, class *Clostridia*, order *Clostridiales*, family *Clostridiaceae*, genus *Clostridium*) is an obligately anaerobic G^+^ sporulating rods [91]. It is one of the primary pathogens producing toxins and causes various diseases of the gastrointestinal tract. *Clostridium difficile* appears to be an important pathogen in patients with IBD and is associated with more severe disease development and increased mortality [107].

In a 2015 study “The impact of *Clostridium difficile* on surgical rate among ulcerative colitis patients: a systematic review and meta-analysis”, the authors evaluated the association between *Clostridium difficile* infection and the rate of colectomy in patients with UC. Statistical analysis was performed using RevMan X6 software (from Cochrane Collaboration) [108].

Patients with UC co-infected with *Clostridium difficile* were found to have statistically significantly higher risks of surgery compared to patients with UC without infection. There was no statistically significant heterogeneity among the included studies (*p* = 0.09). However, the number of studies included was small, so the result should be interpreted with caution [108].

## 7. Conclusions

UC is a chronic disease of the gastrointestinal tract with an as yet unclear etiology. This disease is manifested by an uncontrollable inflammatory immune response. In Europe, around 2.2 million people suffer from UC and the number of patients is still growing. To date, there is no effective treatment to ensure complete recovery of patients. Pharmacotherapy and other procedures only lead to the suppression of symptoms and increase the quality of life of patients, and if this therapy fails, a colectomy may be necessary. Butyrate plays a key role in maintaining intestinal epithelial homeostasis as a preferred source of energy for colonocytes. Butyrate metabolism is one of the current theories for the etiology of UC. It is thought that a lack of energy for colonocytes could lead to the onset of this disease. Butyrate has also been used experimentally in the treatment of colitis with numerous benefits for patients.

SRB are anaerobic bacteria that dissimilate sulfate to H_2_S by a process called sulfate dissimilation reduction. Intestinal SRB include the genera *Desulfotomaculum*, *Desulfobulbus*, *Desulfomicrobium*, *Desulfomonas*, and *Desulfovibrio*. SRB are not thought to be pathogenic in humans and animals, but increased production of toxic H_2_S increases mucosal permeability and blocks butyrate metabolism. UC is a complex topic, connecting a wide range of scientific areas.

Using meta-analysis, we compared data from many scientific papers. Results included an estimate of the effect of treatment or disease risk factors than individual studies. Since 2007, this statistical method has become increasingly popular among scientists. For this research, studies were found that use this method and solve the issue of UC. A meta-analysis from 2020 revealed a lower incidence of IBD within the group of subjects receiving probiotics and at the same time a higher prevalence of IBD in patients with a higher incidence of SRB. Another study evaluated the effectiveness of infliximab treatment. A meta-analysis showed that infliximab reduced the rate of colon colectomy in patients with moderate to severe UC. *Clostridium difficile* appears to be an important pathogen, which has been associated with more severe disease development and increased mortality in patients with IBD. Patients with UC who were co-infected with *Clostridium difficile* were found to have statistically significantly higher risks of surgery compared to patients with UC without infection. Idiopathic intestinal inflammation and assessing the effectiveness of their treatment are extremely urgent topics in biology and medicine. The results of the primary studies are inconsistent, so further analyzes are needed. The meta-analysis appears to be a suitable method for reaching valid conclusions in this area.

## Figures and Tables

**Table 1:** The main representatives of intestinal bacteria [48].

Domain/Phylum	Examples of Genera	Main Function
Phylum: *Bakteroidetes*	*Bacteroidetes*, *Prevotella*, *Xylanibacter*	Degradation complex glycans
Phylum: *Firmicutes*	*Ruminococcus*, *Clostridium*, *Lactobacillus*, *Roseburia*, *Eubacterium*, *Faecalibacterium*	Probiotics, butyrate producers
Phylum: *Actinobacteria*	*Collinsella*, *Bifidobacterium*	Probiotics
Phylum: *Proteobacteria*	*Desulfovibrio*	Sulfate-reducing bacteria
Phylum: *Verrucomicrobia*	*Akkermansia*	Degradation of mucin
Domain: *Archaea*	*Methanobrevibacter*	Methanogenesis

**Table 2:** Examples of representatives of the group of sulfate-reducing organisms [90].

*Archaea*	*Archaeoglobus*, *Caldivirga*, *Vulcanisaeta*	
Bacteria	*Ammonifex*	*Desulfomicrobium*
	*Candidatus*	*Desulfonatronovibrio*
	*Desulfacinum*	*Desulfosarcina*
	*Desulfobacter*	*Desulfosporosinus*
	*Desulfobacterium*	*Desulfotomaculum*
	*Desulfobulbus*	*Desulfovibrio*
	*Desulfocapsa*	*Desulfovirga*
	*Desulfococcus*	*Syntrophobacter*
	*Desulfocurvus*	*Thermodesulfatator*
	*Desulfofustis*	*Thermodesulfobacterium*
	*Desulfohalobium*	*Thermodesulfobium*
	*Desulfoluna*	*Thermodesulfovibrio*

**Table 3:** Perspectives of using meta-analysis for research of ulcerative colitis.

Scientific Discipline	Examples of Potential Use
Medicine and pharmacology	Effectiveness of treatment: infliximab and others medication, probiotics, colectomy
Epidemiology and statistics	Distribution in different parts of the world, age representation of patients in the population, increase or decrease in the number of patients over time
Genetics	Identification of risk genes
Immunology	Etiology of the leaking intestine, etiology of inflammatory reaction
Study of environmental factors	Smoking, stress, diet, influence of other health indications
Microbiology	Relation of microorganisms to UC: sulfate reducing bacteria, methanogenic *archaea*, lactic acid bacteria, *Clostridium difficile*

## Data Availability

Not applicable.
